# CitrusUAT: A dataset of orange *Citrus sinensis* leaves for abnormality detection using image analysis techniques

**DOI:** 10.1016/j.dib.2023.109908

**Published:** 2023-12-07

**Authors:** Wilfrido Gómez-Flores, Juan José Garza-Saldaña, Sóstenes Edmundo Varela-Fuentes

**Affiliations:** a Centro de Investigación y de Estudios Avanzados del Instituto Politécnico Nacional, Ciudad Victoria, Tamaulipas, Mexico; b Facultad de Ingeniería y Ciencias, Universidad Autónoma de Tamaulipas, Ciudad Victoria, Tamaulipas, Mexico

**Keywords:** Citrus diseases, Orange leaves, *Citrus sinensis* (L.) osbeck, Image analysis, Machine learning, Deep learning

## Abstract

Around the world, citrus production and quality are threatened by diseases caused by fungi, bacteria, and viruses. Citrus growers are currently demanding technological solutions to reduce the economic losses caused by citrus diseases. In this context, image analysis techniques have been widely used to detect citrus diseases, extracting discriminant features from an input image to distinguish between healthy and abnormal cases. The dataset presented in this article is helpful for training, validating, and comparing citrus abnormality detection algorithms. The data collection comprises 953 color images taken from the orange leaves of *Citrus sinensis* (L.) Osbeck species. There are 12 nutritional deficiencies and diseases supporting the development of automatic detection methods that can reduce economic losses in citrus production.

Specifications Table

**Table:** 

Subject	Agricultural Sciences and Computer Science
Specific subject area	Agriculture Engineering, Digital Image Analysis, Machine Learning
Data format	Conversion: JPG Annotations: PNG
Type of data	Image
Data collection	Orange leaf samples of *Citrus sinensis* (L.) Osbeck species with nutritional deficiencies, diseases, and pest symptoms were collected and photographed using a color camera and a portable studio. Next, samples were analyzed by the quantitative real-time polymerase chain reaction (qPCR) diagnostic test to detect the *Ca.* L. asiaticus bacterium that causes Huanglongbing disease. Besides, each color image was segmented by a thresholding method to obtain a binary mask of the leaf region.
Data source location	Orange groves in the States of Tamaulipas and San Luis Potosi, Mexico
Data accessibility	https://doi.org/10.5281/zenodo.8294078
Related research article	Wilfrido Gómez-Flores, Juan José Garza-Saldaña, and Sóstenes Edmundo Varela-Fuentes, A Huanglongbing Detection Method for Orange Trees Based on Deep Neural Networks and Transfer Learning, IEEE Access, vol. 10, pages 116686-116696, 2022. DOI:10.1109/ACCESS.2022.3219481

## 1 Value of the Data


 

• This dataset provides color images of the orange leaves of *Citrus sinensis* (L.) Osbeck species with nutritional deficiencies, diseases, and pest symptoms.
• The data collection comprises 953 color images divided into 12 classes of orange leaves.
• The dataset includes ground-truth binary masks for each image to identify regions of interest for citrus abnormality classification.
• The quantitative real-time polymerase chain reaction (qPCR) test analyzed the leaf samples to detect the *Ca.* L. asiaticus bacterium that causes Huanglongbing disease.
• The image dataset is helpful for training, validating, and comparing citrus disease detection methods based on image analysis and machine learning.
• Researchers and professionals interested in automatically detecting citrus disorders can use this dataset.

## 2 Objective

Citrus is the second most harvested fruit in the world [1]. However, there are geographic regions where citrus production and quality are threatened by diseases caused by fungi, bacteria, pests, and viruses [2]. Hence, to reduce economic losses, citrus growers are looking for technologies capable of detecting citrus abnormalities automatically and accurately. The purpose of this dataset is to provide a collection of color images taken from the orange leaves of *Citrus sinensis* (L.) Osbeck species with diseases, nutritional deficiencies, and pest symptoms, proper to develop abnormality detection algorithms based on digital image analysis techniques. The dataset is divided into 12 classes of orange leaves: Healthy, Huanglongbing (HLB), Greasy spot, Iron deficiency, Magnesium deficiency, Manganese deficiency, Nitrogen deficiency, Zinc deficiency, Texas citrus mite, Red scale, Red scale sequelae, and Citrus leafminer [3].

HLB is relevant in this dataset since it is one of the most destructive citrus diseases. The suspicious causal pathogen of this disease is a bacterium of the genus *Candidatus* Liberibacter—specifically, *Ca*. L. asiaticus has been identified in the northeast of Mexico and is transmitted by the Asian citrus psyllid *Diaphorina citri* [4]. Currently, HLB has no cure and may coexist with other citrus abnormalities whose symptoms could overlap with HLB. Consequently, it is crucial to distinguish between HLB and different kinds of disorders to apply adequate treatment and sanitary protocol [5]. In this regard, an HLB-infected tree is removed to avoid spreading the disease in the orchard. On the other hand, if a tree has a nutritional deficiency, pest, or some other curable disease, a remedial treatment is applied.

## 3 Data Description

**Fig. 1:**
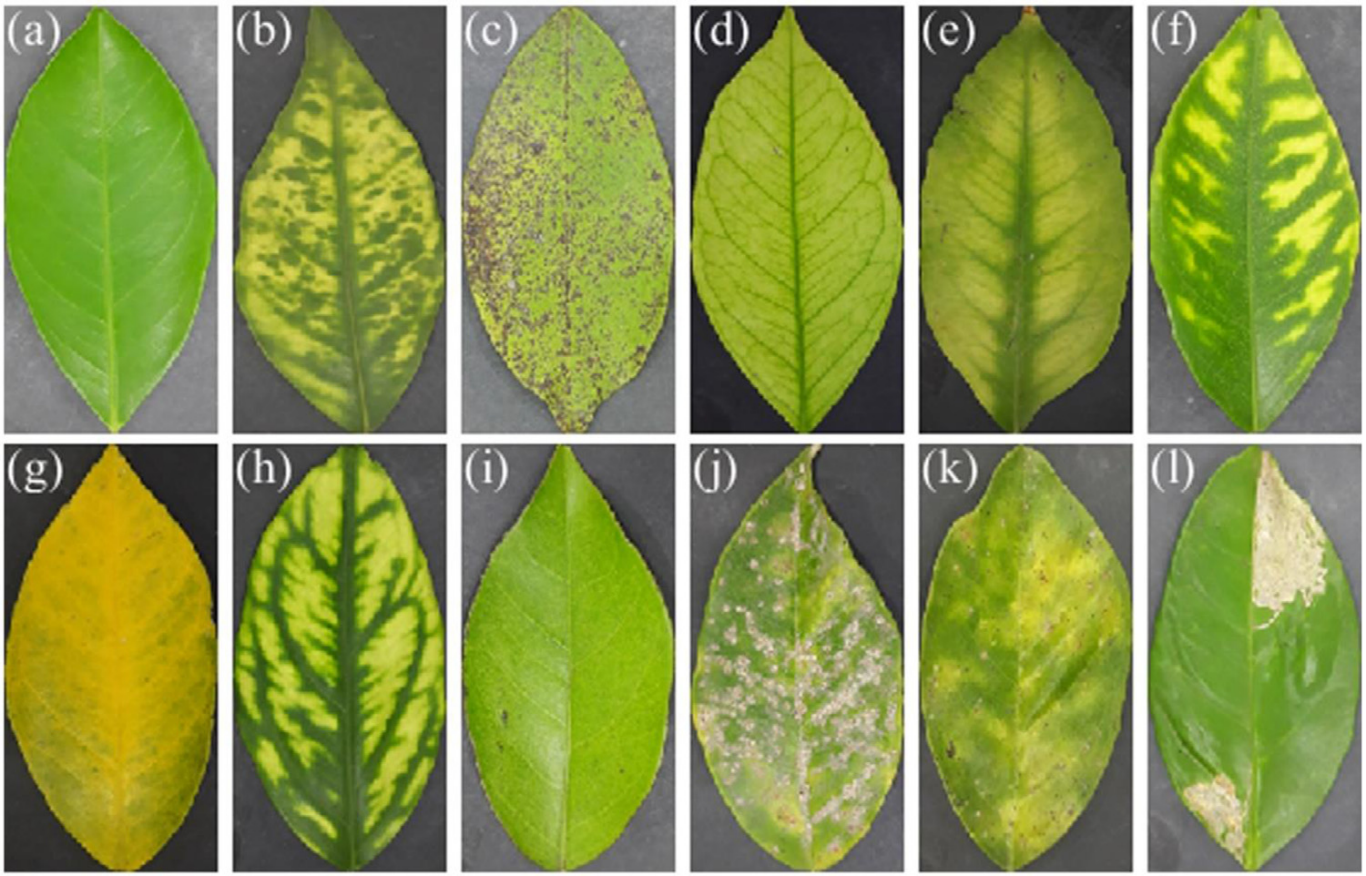
Leaf samples in the dataset: (a) Healthy, (b) Huanglongbing, (c) Greasy spot, (d) Fe-deficiency, (e) Mg-deficiency, (f) Mn-deficiency, (g) N-deficiency, (h) Zn-deficiency, (i) Texas citrus mite, (j) Red scale, (k) Red scale sequelae, and (l) Citrus leafminer.

The dataset comprises 953 color images of orange leaves of the *Citrus sinensis* (L.) Osbeck species, collected in orange groves in the states of Tamaulipas and San Luis Potosi in the northeast of Mexico. Table 1 shows the 12 classes of orange leaves in the dataset, including healthy, diseased, nutrient deficient, and pests. Additionally, Fig. 1 shows representative samples of each abnormality, illustrating the expected differences in texture and color patterns of the leaves [3].

**Table 1:** Class-wise image distribution in the dataset.

Class name	# Images	Abnormality type
Healthy	100	Not abnormal
HLB	43	Disease
Greasy spot	100	Disease
Fe	100	Iron deficiency
Mg	100	Magnesium deficiency
Mn	30	Manganese deficiency
N	50	Nitrogen deficiency
Zn	100	Zinc deficiency
Texas mite	100	Pest
Red scale	30	Pest
Red scale sequelae	100	Pest
Citrus leafminer	100	Pest

The primary directory is named “CitrusUAT_dataset” and contains the following three subdirectories with 953 files each:

1. Images: original color images of orange leaves in JPG format.
2. Masks: binary masks of segmented leaves in PNG format.
3. ROIs: binary masks of circular regions of interest (ROIs) in PNG format.

**Fig. 2:**
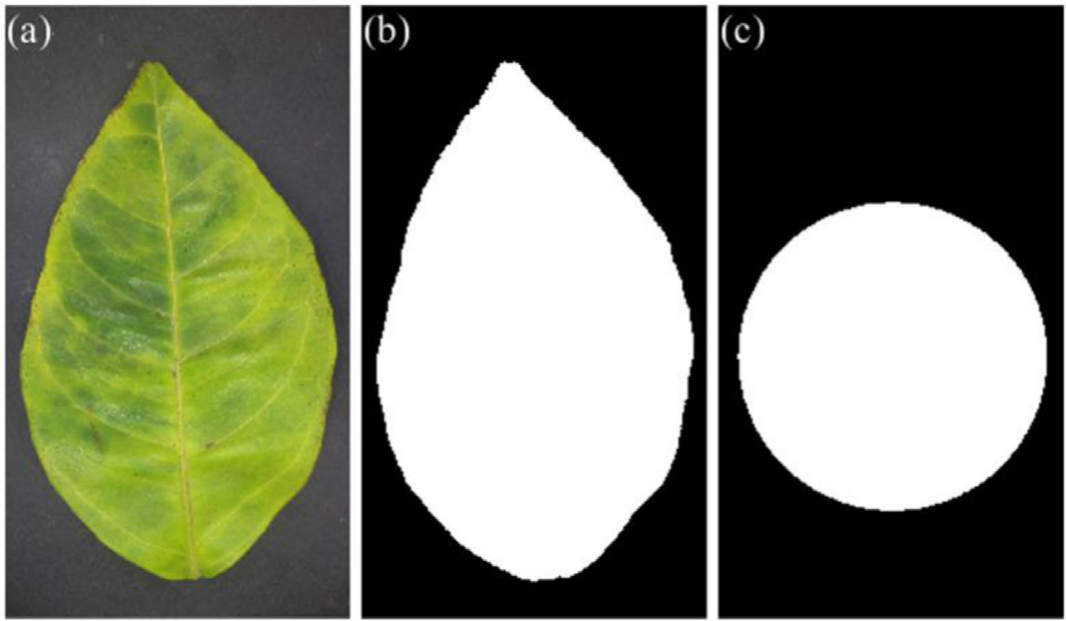
Images from the Images, Masks, and ROIs folders: (a) color image from the HLB_001.jpg file, (b) leaf binary mask from the HLB_001_mask.png file, and (c) ROI binary mask from the HLB_001_roi.png file.

The name of each file in the “Images” folder has the following syntax: “Abnormality_Number.jpg”, where “Abnormality” is one of the class names given in Table 1, and “Number” is the sample number for its respective class; for instance, “HLB_001.jpg” means that the image has the first sample of the HLB class. Concerning the PNG files in the “Masks” and “ROIs” folders, the suffixes “mask” and “roi” are attached to the name of their corresponding image in the “Images” folder; for instance, “HLB_001_mask.png” and “HLB_001_roi.png”. The size of all the images is 4128 × 3096 pixels. Fig. 2 shows examples of the color image and binary masks for the first sample of the HLB class.

## 4 Experimental Design, Materials, and Methods

### 4.1 Dataset acquisition

Experts of the State Plant Health Committee of Tamaulipas (CESAVETAM, Mexico) collected and identified abnormalities in orange groves. This activity was carried out between August 2016 and March 2019. The protocol to obtain the image dataset consists of the following steps:

1. Cut leaf samples in full development from four branches of the orange tree.
2. Take color images of samples with a conventional camera using a portable studio with controlled lighting and a homogeneous background.
3. Place the samples on absorbent paper towels and put them in sealed plastic bags with their corresponding identification number.
4. Transport the samples in ice chests to the Molecular Detection Laboratory (Empacadora Santa Engracia, Tamaulipas, Mexico).
5. Detect the bacterium *Ca.* L. asiaticus in leaf samples using quantitative polymerase chain reaction (qPCR) analysis to confirm HLB, following the National Phytosanitary Reference Center protocol of the General Directorate of Plant Health (CNRF, Mexico) [6].

**Fig. 3:**
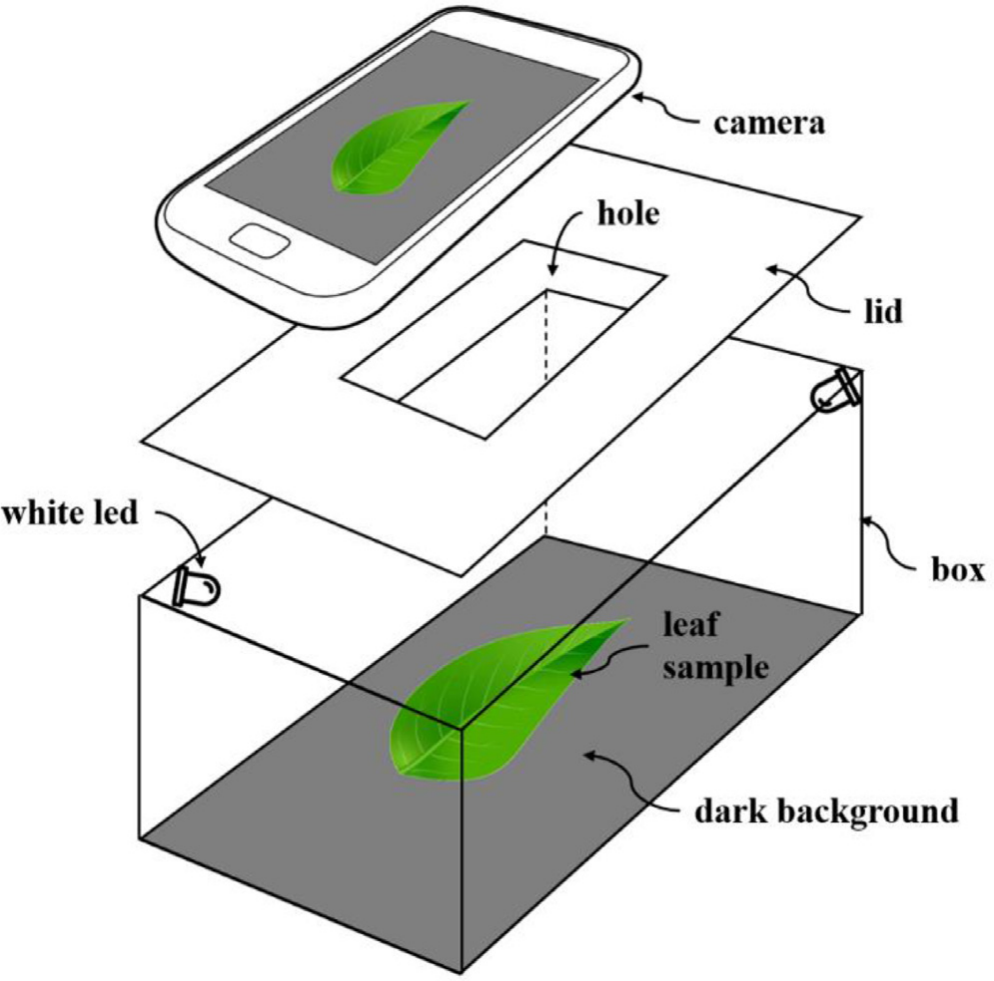
The portable studio for in-field image acquisition. The leaf is placed on the bottom of a box illuminated with artificial light. The base is dark to avoid reflections. The lid of the box has a hole for the lens of a camera.

In the second step of the collection protocol, the samples were photographed *in situ* using a portable studio, shown in Fig. 3. Every leaf sample is placed on the dark bottom of the box, which is internally illuminated by white LEDs. The box is closed with a lid with a hole for the camera lens. This portable mounting has two purposes. First, to control the illumination conditions of in-field image acquisition. Second, because the bottom of the box is dark, the color of the leaf is well contrasted so that the segmentation method is simplified to the automatic search of a global intensity threshold, as detailed elsewhere in this paper. The pictures were shot with a Samsung Galaxy phone (Samsung Electronics, Suwon, South Korea), model SM-J730 Pro, with 13 megapixels, without zoom and flash, and saved in JPG format.

### 4.2 Leaf segmentation

The original color image is automatically segmented to obtain a binary mask of the leaf shape. The segmentation method is composed of the following steps [3]:

1. Convert the image from RGB to LAB colorspace for obtaining the luminance and chrominance components.
2. Separate the chromatic B component and reduce the noise with a Gaussian filter with a kernel size of 5 × 5 pixels.
3. Apply an intensity threshold, given by Otsu's method, to obtain the segmented leaf region.

**Fig. 4:**
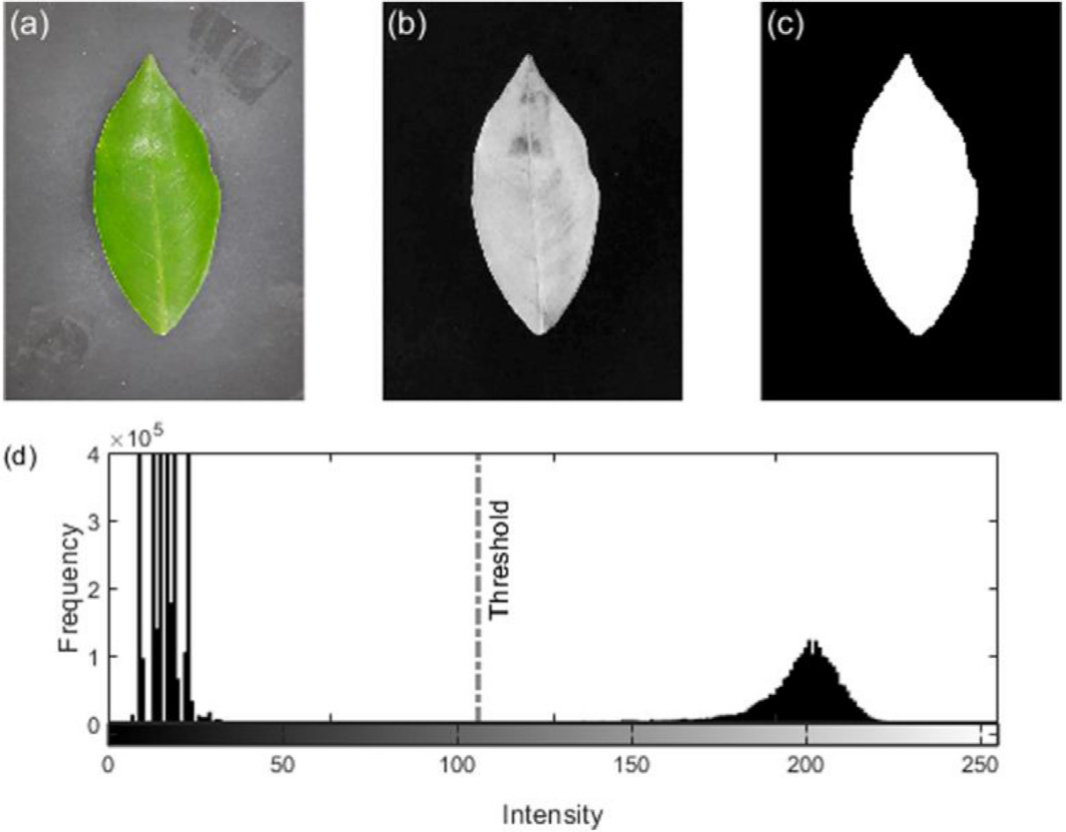
(a) Original RGB image, (b) B component from LAB colorspace, (c) segmented leaf (binary mask), and (d) intensity histogram of the B component; the dashed line marks the intensity threshold to obtain the binary image in (c).

Fig. 4 shows a leaf image segmented by this method. The chromatic B component ensures the bimodality of the intensity histogram to segment the leaf shape by using a threshold given by Otsu's method [7].

### 4.3 Circular region of interest

The segmentation method described above defines the entire region occupied by the leaf over the image plane. However, in some image analysis procedures, it is necessary to extract features from the internal region of the leaf. A region of interest (ROI) with the largest area within the leaf was obtained, aiming to extract the most pixel information. Besides, the aspect ratio of the texture patterns within the ROI should be preserved when rescaling the ROI's size, for instance, when using convolutional neural networks with a fixed-sized input layer. Hence, the most natural shape is a circular ROI to cope with these specifications. The steps for extracting the maximum inscribed circle within the leaf region are [3]:

1. Calculate the distance map of the binary mask of the leaf shape.
2. Get the maximum distance value $d_{max}$ in the distance map.
3. Extract the (x,y) coordinates in the distance map with values equal to $d_{max}$.
4. Average the (x,y) coordinates to get the centroid $\left(\bar{x},\bar{y}\right)$ of the circular ROI.
5. Calculate the Euclidean distance from the point $\left(\bar{x},\bar{y}\right)$ to all pixels in the image.
6. Obtain the circular ROI by binarizing the Euclidean distance map in step 5 using $d_{max}$ as a threshold.

**Fig. 5:**
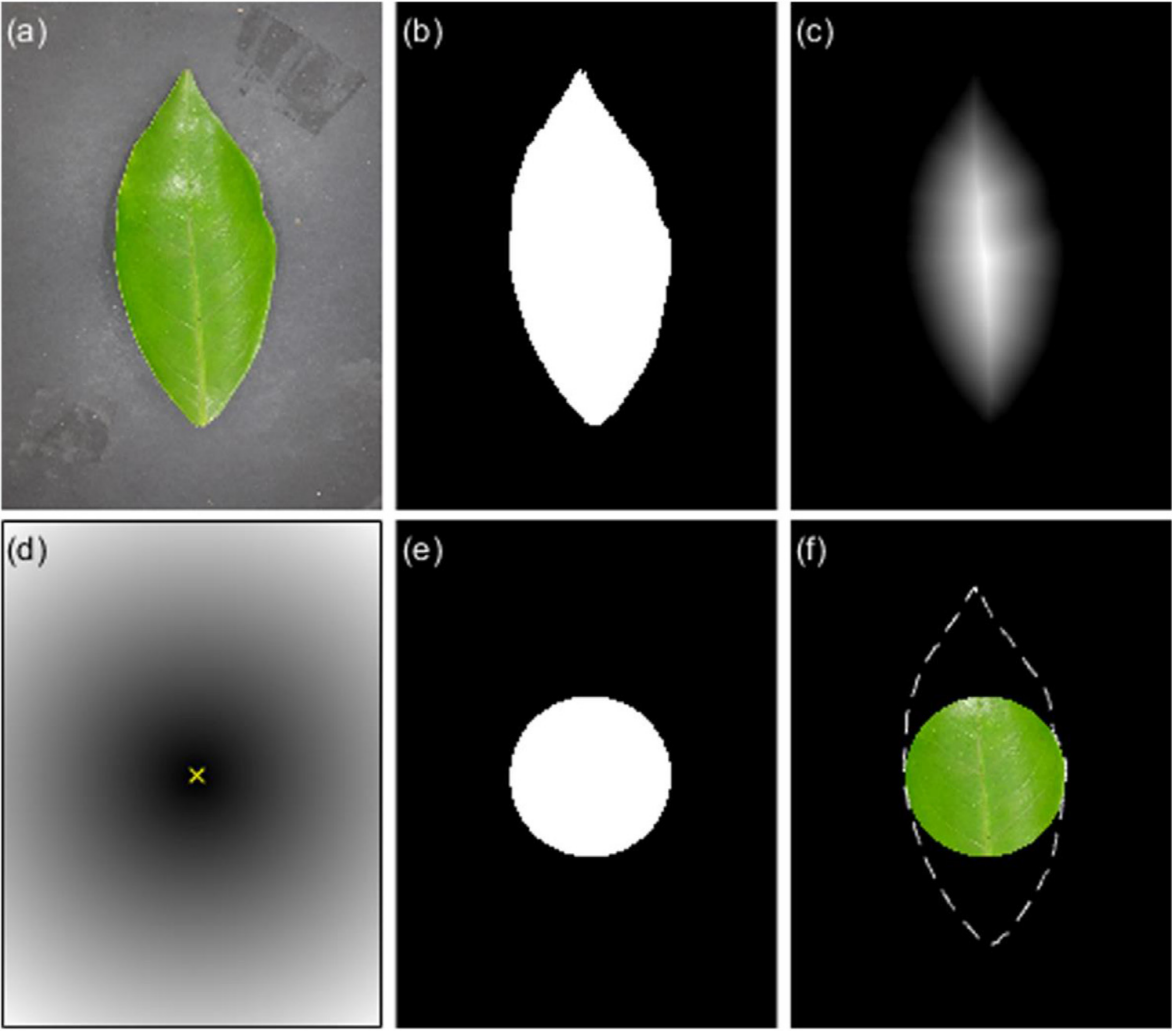
(a) Original color image, (b) binary mask of the segmented leaf, (c) distance map of leaf region, (d) Euclidean distance from the centroid (× symbol) to all pixels, (e) circular ROI mask, and (f) masked original image, the dashed contour is a visual reference of the leaf shape.

Fig. 5 shows the circular ROI obtained with the previously described method. Note that the original image is masked to get only pixel information from the inner region of the leaf.

### 4.4 Croping images using binary masks

**Fig. 6:**
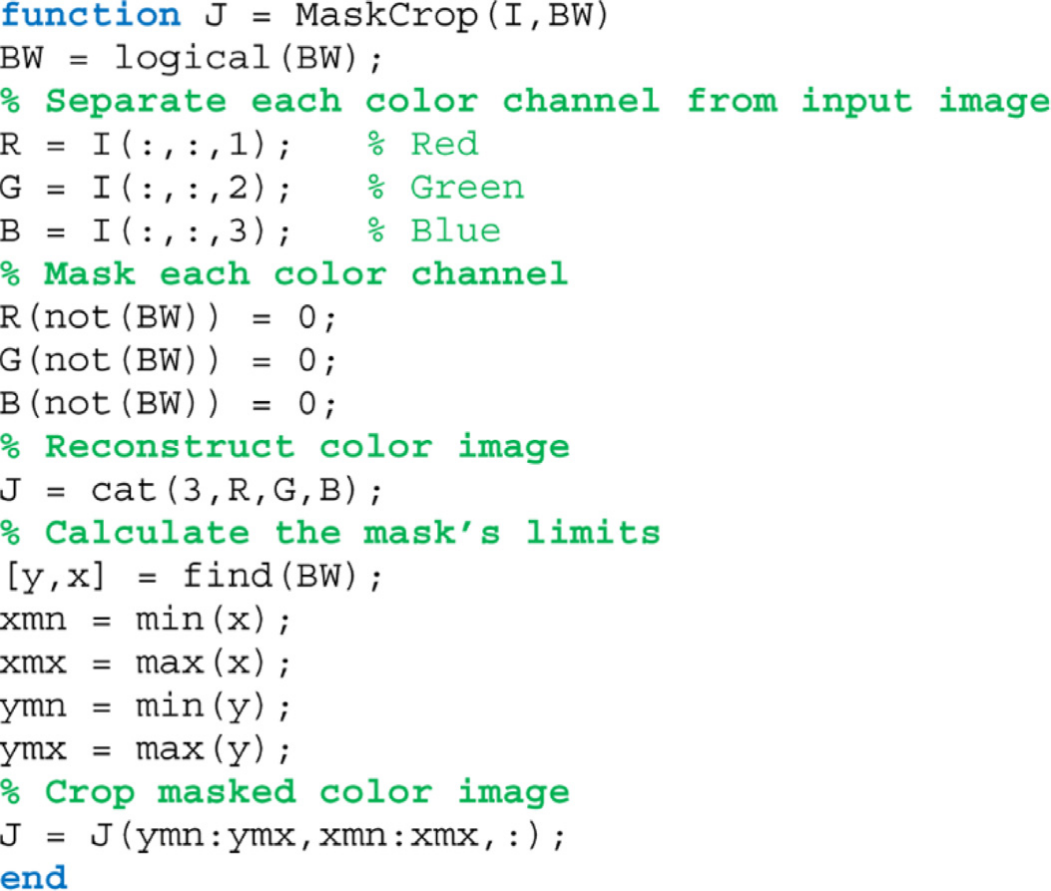
The MATLAB function to crop a leaf image using a binary mask.

**Fig. 7:**
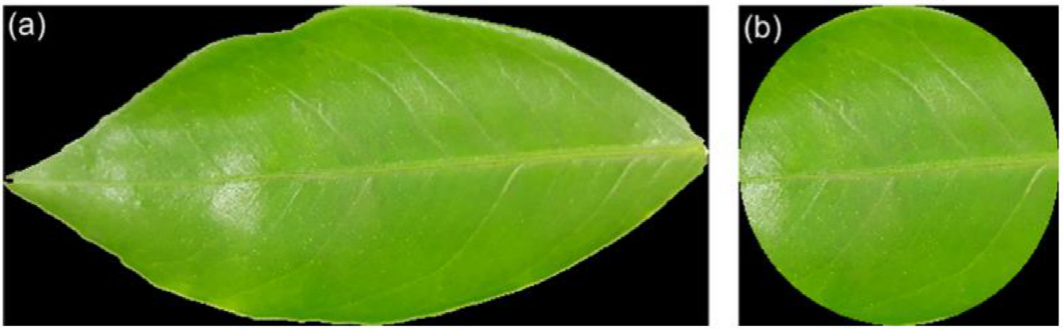
Original color image cropped with (a) binary mask of the segmented leaf and (b) binary mask of the circular ROI.

The binary masks of the segmented leaf shape and the circular ROI are useful for cropping the color image before using image analysis techniques. This way, unuseful pixels are removed to reduce the computing time of the algorithms. Fig. 6 shows the MATLAB code to mask and crop a color image using a binary mask, from which the images in Fig. 7 are obtained.

### 4.5 Artificial data augmentation recommendations

Currently, citrus disease classification is commonly performed by convolutional neural networks (CNNs) since the data representations are automatically learned by transforming the input image into multiple layers of abstractions [8], [9], [10], [11]. CNN architectures usually have millions of trainable parameters, making CNNs prone to overfitting when training from scratch using a small dataset. This issue is because the more learnable parameters, the higher the possibility that activation patterns specific to examples in the training set will happen. Hence, CNN training requires a large volume of annotated data [12].

Collecting many annotated data was unfeasible in our dataset, mainly because the qPCR test to detect HLB was costly, requiring the services of a specialized laboratory. Hence, our previous study used artificial data augmentation to reduce deep neural network overfitting [3], and we recommend it when training CNN-based applications.

Artificial data augmentation increases the quantity of data by randomly applying image transformations to the training set [11]. This implicit regularization reduces overfitting because it is unlikely to generate the same image twice, enabling invariance and adding an extra source of stochasticity to CNN learning [13].

**Fig. 8:**
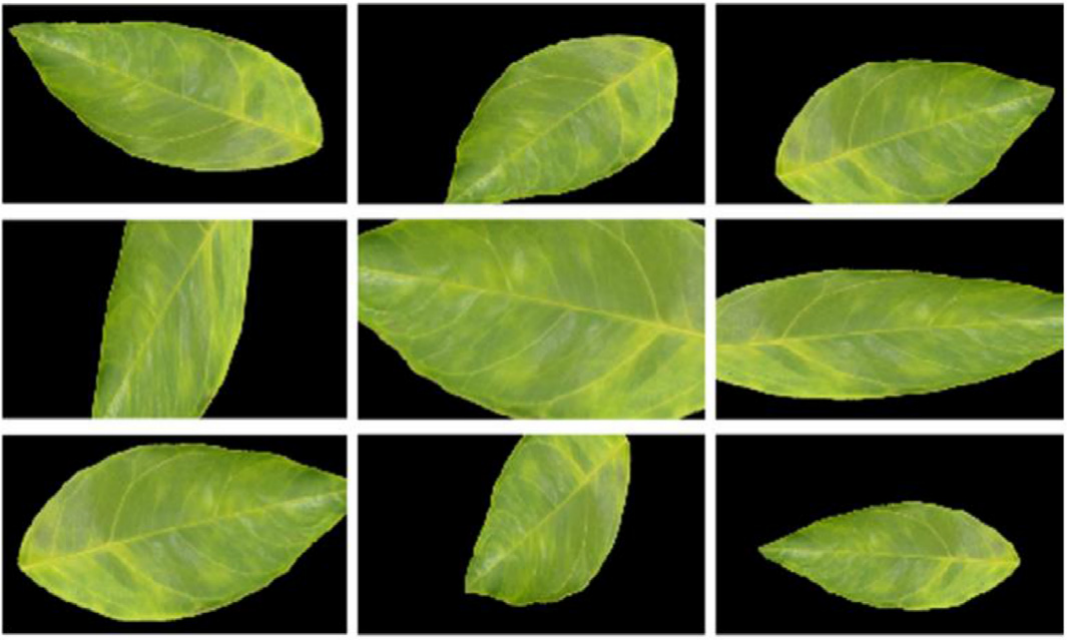
Artificial data augmentation of an HLB-infected case.

**Fig. 9:**
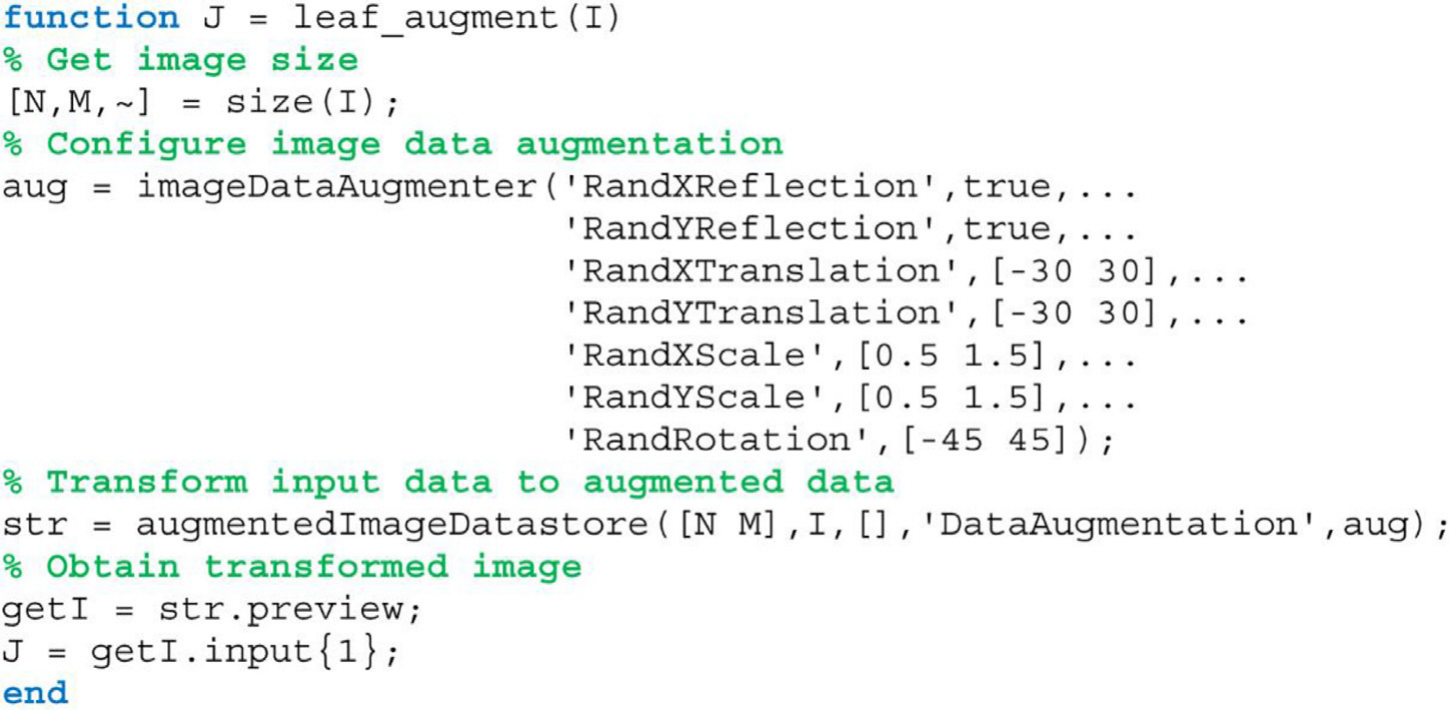
The MATLAB function for artificial data augmentation. It requires the built-in functions imageDataAugmenter and augmentedImageDatastore of the Deep Learning toolbox.

We recommend using online data augmentation, i.e., the augmented images are generated on the fly at training time by randomly applying geometric transformations to the mini-batches [13]. The advantage is that the generated images are not physically stored on disk, reducing computing hardware resources. Table 2 summarizes the recommended geometric transformations and their operative ranges. Besides, Fig. 8 shows examples of artificial data augmentation generated with the MATLAB code in Fig. 9.

**Table 2:** Artificial data augmentation parameters.

Transformation	Range
Reflection	Vertical and horizontal
Translation *x*- and *y*-axes	[−30, 30] pixels
Scaling *x*- and *y*-axes	[0.5, 1.5]
Rotation	[−45°, 45°]

It is worth mentioning that artificial data augmentation can also be used to increase the number of cases in unbalanced classes, avoiding classification performance biased to the majority class [14].

### 4.6 Comparison with public citrus disease datasets

The PlantVillage image dataset has been widely used to develop disease detection systems for various plants such as apples, cherries, grapes, oranges, and tomatoes [15]. However, this dataset only includes HLB-infected leaf samples without considering other citrus diseases. In contrast, our dataset considers different classes of orange leaves, aiming to include other orange disorders that may coexist with HLB in the orchard.

On the other hand, the Citrus-Plant dataset is specific for citrus diseases [16]. It contains 759 images of citrus leaves and fruits with five classes of abnormalities (HLB included) and healthy ones. Nevertheless, the CitrusUAT dataset contains 953 leaf images and 12 classes, augmenting the variety of orange leaf abnormalities.

Additionally, HLB cases in our dataset were diagnosed by qPCR to confirm the presence of *Ca*. L. asiaticus in oranges of *Citrus sinensis* (L.) Osbeck species, which is not clarified in PlantVillage and Citrus-Plant datasets. Indeed, some HLB annotated cases from the PlantVillage dataset are not typical HLB symptoms or are probably caused by other diseases, so they are dropped in some approaches [17]. Hence, our dataset guarantees the correct identification of HLB analogous to the biopsy exam in cancer applications. Besides, the CitrusUAT dataset includes binary masks for all the images, which is useful for semantic segmentation tasks.

## Limitations

None.

## Ethics Statement

The proposed dataset does not involve human subjects, animal experiments, or data collected from social media platforms.

## CRediT authorship contribution statement

**Wilfrido Gómez-Flores:** Conceptualization, Methodology, Software, Writing – review & editing. **Juan José Garza-Saldaña:** Resources, Data curation, Methodology, Writing – original draft. **Sóstenes Edmundo Varela-Fuentes:** Supervision, Funding acquisition.

## Data Availability

CitrusUAT: A Dataset of Orange Citrus sinensis Leaves for Abnormality Detection Using Image Analysis Techniques (Original data) (Zenodo).
